# The genome sequence of the Pearl-bordered Fritillary, *Boloria euphrosyne *(Linnaeus, 1758)

**DOI:** 10.12688/wellcomeopenres.21586.1

**Published:** 2024-05-15

**Authors:** Simon H. Martin, Konrad Lohse, Sam Ebdon, Alex Mackintosh, Wellcome Sanger Institute Tree of Life Management, Samples and Laboratory team, Wellcome Sanger Institute Scientific Operations: Sequencing Operations, Wellcome Sanger Institute Tree of Life Core Informatics team, Tree of Life Core Informatics collective, Darwin Tree of Life Consortium

**Affiliations:** 1 Institute of Ecology and Evolution, The University of Edinburgh, Edinburgh, Scotland, UK

**Keywords:** Boloria euphrosyne, Pearl-bordered Fritillary, genome sequence, chromosomal, Lepidoptera

## Abstract

We present a genome assembly from an individual male *Boloria euphrosyne* (the Pearl-bordered Fritillary; Arthropoda; Insecta; Lepidoptera; Nymphalidae). The genome sequence is 400.4 megabases in span. Most of the assembly is scaffolded into 31 chromosomal pseudomolecules, including the Z sex chromosome. The mitochondrial genome has also been assembled and is 15.17 kilobases in length. Gene annotation of this assembly on Ensembl identified 19,138 protein coding genes.

## Species taxonomy

Eukaryota; Opisthokonta; Metazoa; Eumetazoa; Bilateria; Protostomia; Ecdysozoa; Panarthropoda; Arthropoda; Mandibulata; Pancrustacea; Hexapoda; Insecta; Dicondylia; Pterygota; Neoptera; Endopterygota; Amphiesmenoptera; Lepidoptera; Glossata; Neolepidoptera; Heteroneura; Ditrysia; Obtectomera; Papilionoidea; Nymphalidae; Heliconiinae; Argynnini; *Boloria*; *Boloria euphrosyne* (Linnaeus, 1758) (NCBI:txid405023).

## Background

The Pearl-bordered Fritillary ( *Boloria euphrosyne*) is a butterfly in the family Nymphalidae. It is found throughout much of the palearctic region, but is restricted to woodland clearings, bracken/scrub/grassland, and open woodland habitats ( Eeles, 2019). Its larval host plants are *Viola* species. Adults appear in April or May depending on locality, with a single brood per year in more northerly parts of the range and two broods per year in more southerly parts ( Eeles, 2019).

*Boloria euphrosyne* was historically one of the UK’s most common woodland butterflies and is classed as a species of Least Concern on IUCN Red list ( Van Swaay *et al.*, 2010). However, its numbers have declined dramatically in the UK during the past century ( Fox *et al.*, 2015), and it is now classed as Vulnerable on the UK Red List ( Fox *et al.*, 2022). These declines have been attributed to loss of suitable habitat – specifically early successional habitats, where it oviposits on young *Viola* seedlings ( Greatorex-Davies *et al.*, 1992). Such suitable habitats have diminished due to a reduction in coppicing practices.

It has been suggested that the dependence UK populations of *B. euphrosyne* on man-made habitats indicates that the butterfly is a relic from a warmer period, with recently coppiced areas providing a warm refuge for extant populations ( Thomas, 1993). Indeed, unlike its *Viola*-feeding congener *Boloria selene*, *B. euphrosyne* shows strong preference for warm microclimates during egg-laying, consistent with a high temperature sensitivity ( Wilby *et al.*, 2024). Paradoxically, the warming climate may be accelerating declines of *B. euphrosyne* in the UK by causing increased vegetation cover and cooler microclimates around their host plants ( Wilby *et al.*, 2024).

The Pearl-bordered Fritillary has 31 chromosomes ( Lorković, 1941; Robinson, 1971). We present here a chromosomal-level genome sequence of *Boloria euphrosyne*, generated as part of the Darwin Tree of Life project. This assembly will be valuable for genomic studies into diversity and adaptation in this species, as well as for evolutionary analyses of the genus *Boloria*.

## Genome sequence report

**Figure 1.**
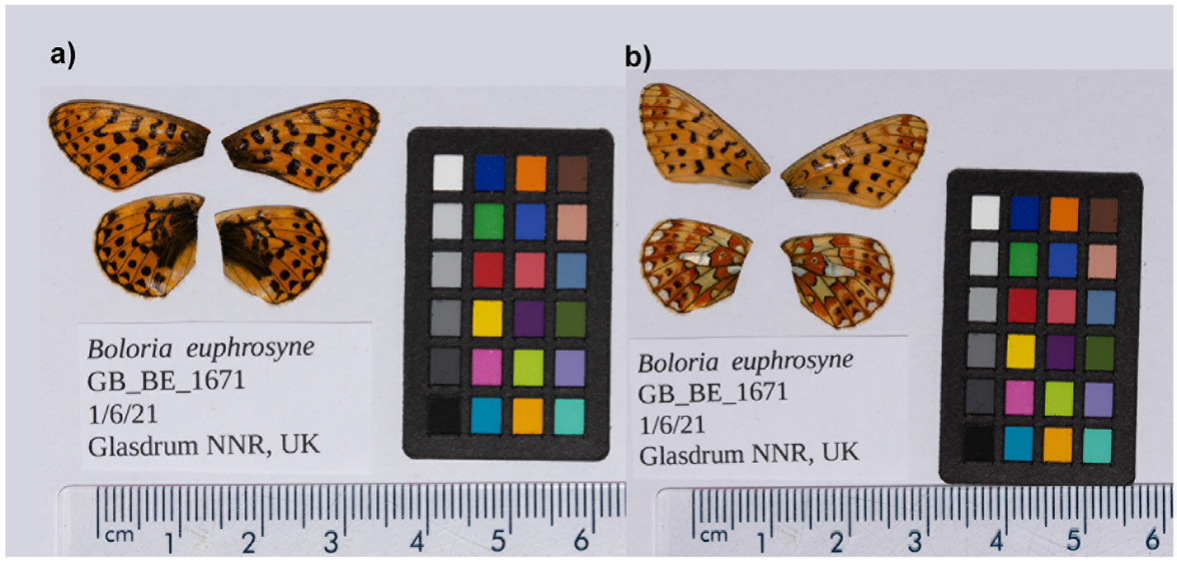
Photographs of forewings and hindwings of the *Boloria euphrosyne* specimen GB_BE_1671 used for genome sequencing: **a**) Dorsal and **b**) ventral surface views of wings from the specimen.

The genome was sequenced from a male *Boloria euphrosyne* ( Figure 1) collected from Glasdrum National Nature Reserve, Scotland, UK (56.57, –5.23). A total of 65-fold coverage in Pacific Biosciences single-molecule HiFi long reads was generated. Primary assembly contigs were scaffolded with chromosome conformation Hi-C data. Manual assembly curation corrected 9 missing joins or mis-joins and removed 2 haplotypic duplications.

**Figure 2.**
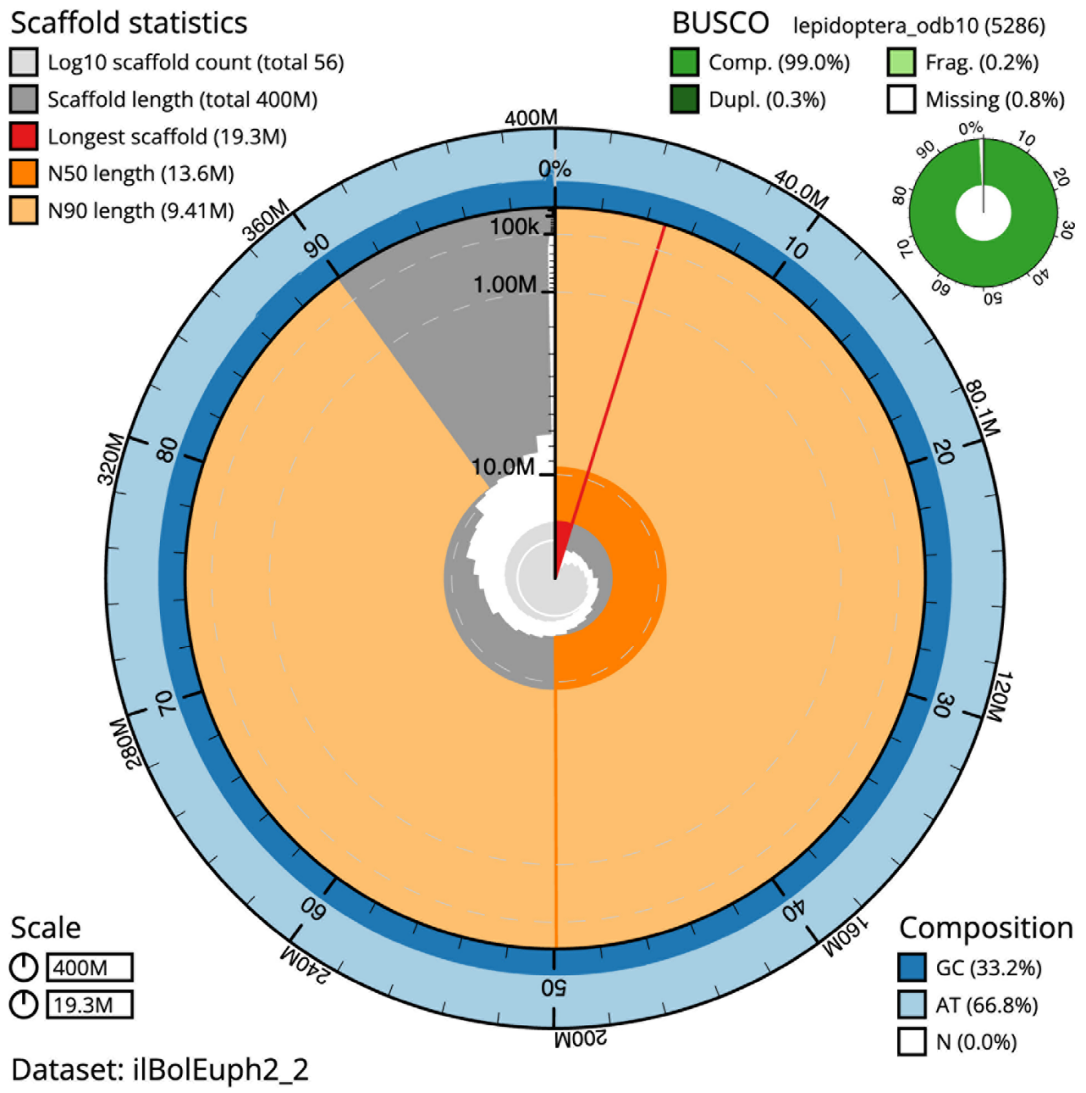
Genome assembly of *Boloria euphrosyne*, ilBolEuph2.2: metrics. The BlobToolKit snail plot shows N50 metrics and BUSCO gene completeness. The main plot is divided into 1,000 size-ordered bins around the circumference with each bin representing 0.1% of the 400,451,666 bp assembly. The distribution of scaffold lengths is shown in dark grey with the plot radius scaled to the longest scaffold present in the assembly (19,276,277 bp, shown in red). Orange and pale-orange arcs show the N50 and N90 scaffold lengths (13,609,687 and 9,410,304 bp), respectively. The pale grey spiral shows the cumulative scaffold count on a log scale with white scale lines showing successive orders of magnitude. The blue and pale-blue area around the outside of the plot shows the distribution of GC, AT and N percentages in the same bins as the inner plot. A summary of complete, fragmented, duplicated and missing BUSCO genes in the lepidoptera_odb10 set is shown in the top right, An interactive version of this figure is available at https://blobtoolkit.genomehubs.org/view/ilBolEuph2_2/dataset/ilBolEuph2_2/snail.

**Figure 3.**
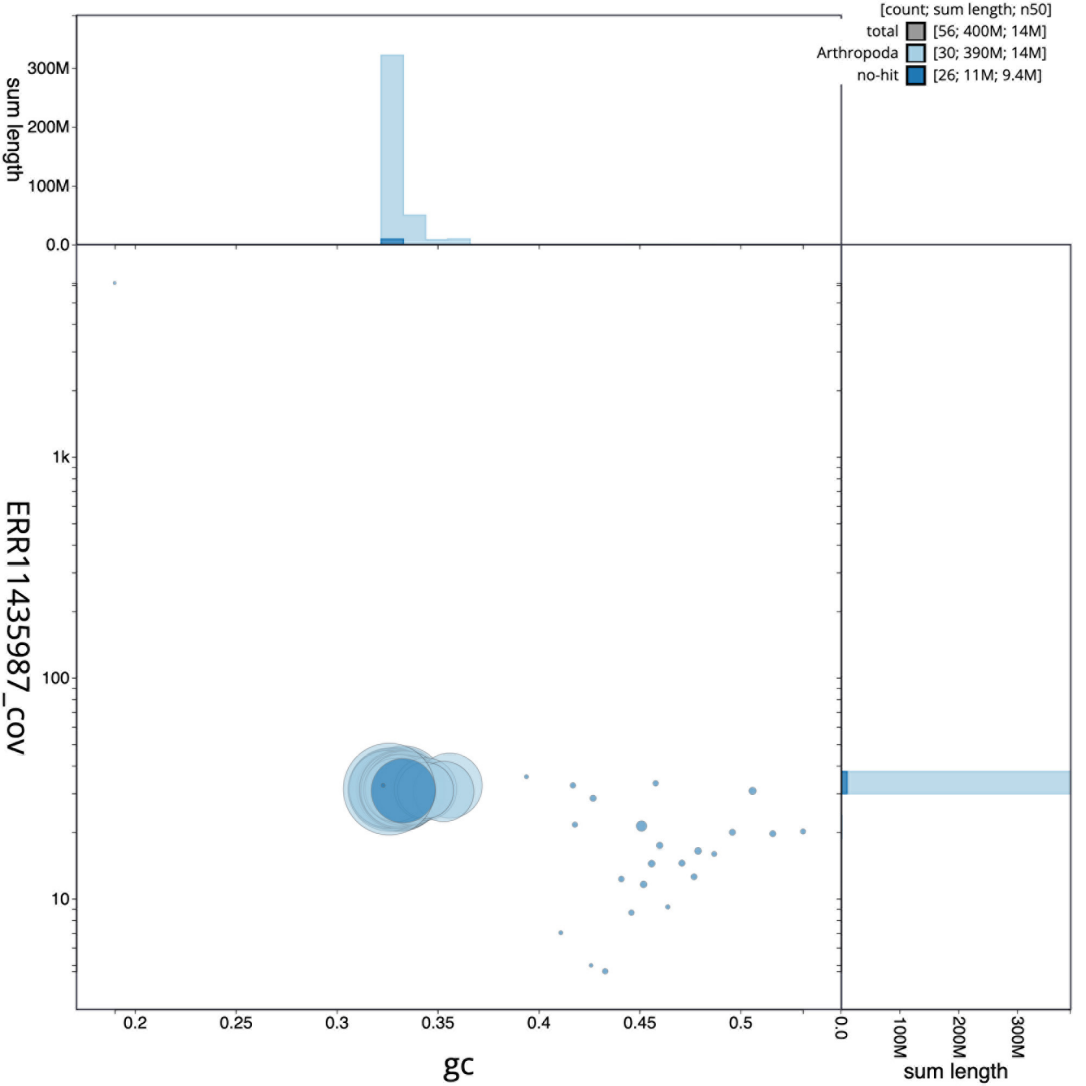
Genome assembly of *Boloria euphrosyne*, ilBolEuph2.2: BlobToolKit GC-coverage plot. Sequences are coloured by phylum. Circles are sized in proportion to sequence length. Histograms show the distribution of sequence length sum along each axis. An interactive version of this figure is available at https://blobtoolkit.genomehubs.org/view/ilBolEuph2_2/dataset/ilBolEuph2_2/blob.

**Figure 4.**
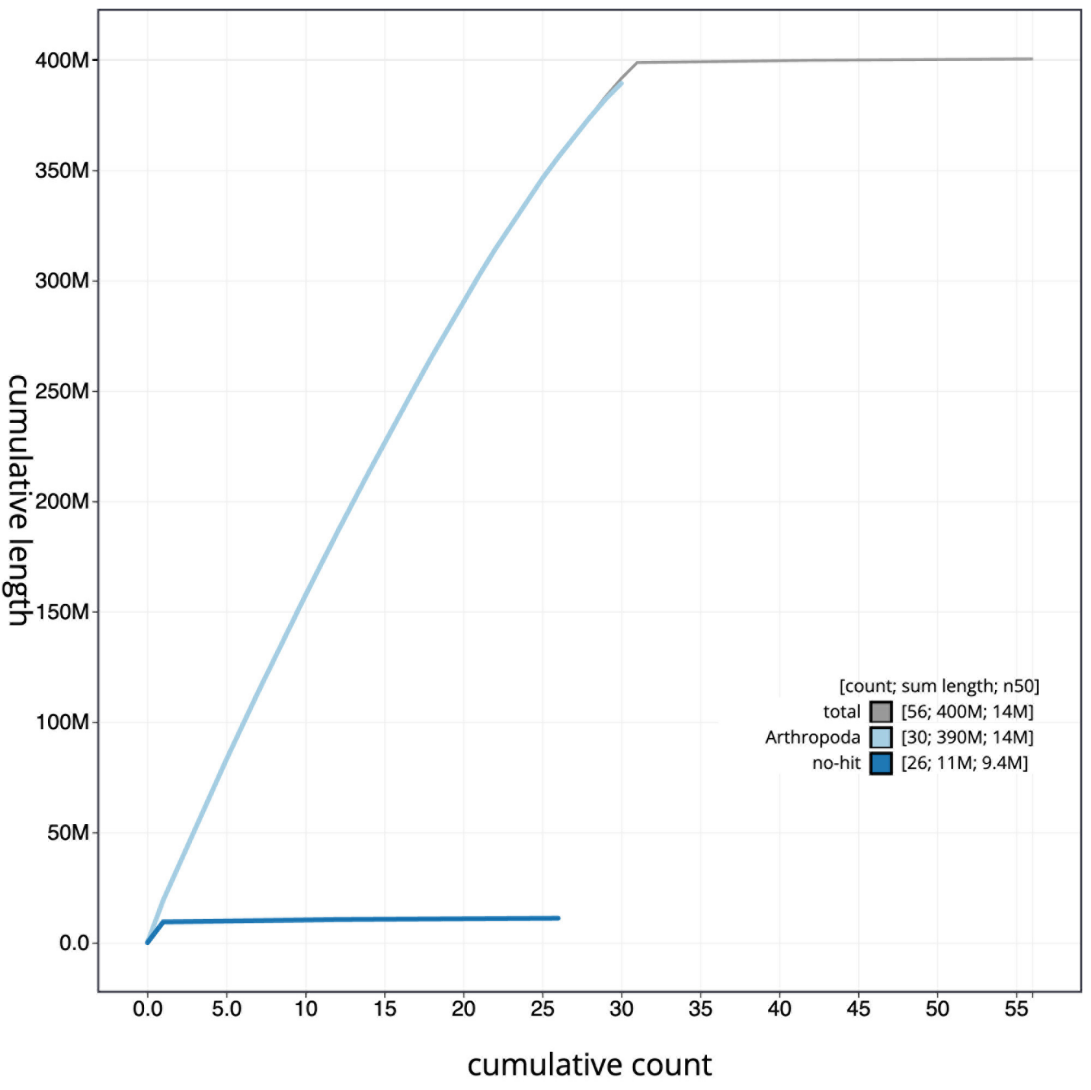
Genome assembly of *Boloria euphrosyne*, ilBolEuph2.2: BlobToolKit cumulative sequence plot. The grey line shows cumulative length for all sequences. Coloured lines show cumulative lengths of sequences assigned to each phylum using the buscogenes taxrule. An interactive version of this figure is available at https://blobtoolkit.genomehubs.org/view/ilBolEuph2_2/dataset/ilBolEuph2_2/cumulative.

**Figure 5.**
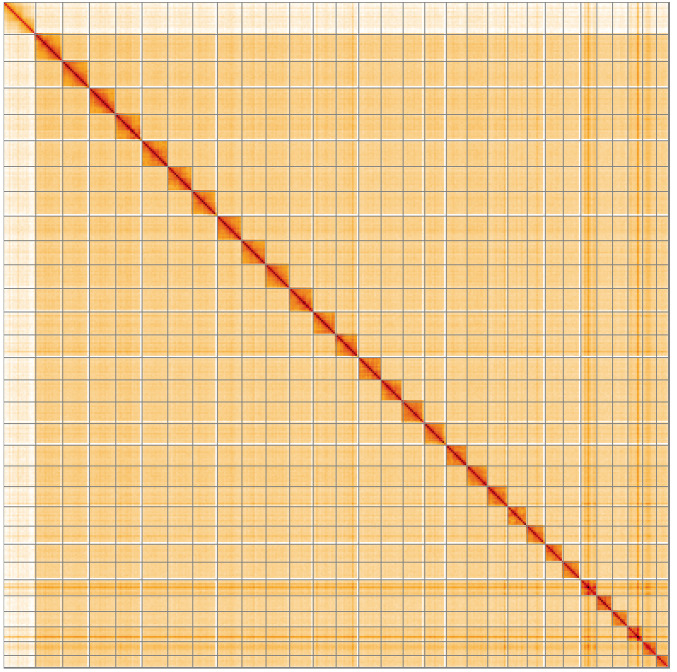
Genome assembly of *Boloria euphrosyne*, ilBolEuph2.2: Hi-C contact map of the ilBolEuph2.2 assembly, visualised using HiGlass. Chromosomes are shown in order of size from left to right and top to bottom. An interactive version of this figure may be viewed at https://genome-note-higlass.tol.sanger.ac.uk/l/?d=fsUukdgOTl23VxxfbdsYYA.

The final assembly has a total length of 400.4 Mb in 55 sequence scaffolds with a scaffold N50 of 13.6 Mb ( Table 1). The snail plot in Figure 2 provides a summary of the assembly statistics, while the distribution of assembly scaffolds on GC proportion and coverage is shown in Figure 3. The cumulative assembly plot in Figure 4 shows curves for subsets of scaffolds assigned to different phyla. Most (99.57%) of the assembly sequence was assigned to 31 chromosomal-level scaffolds, representing 30 autosomes and the Z sex chromosome. Chromosome-scale scaffolds confirmed by the Hi-C data are named in order of size ( Figure 5; Table 2). While not fully phased, the assembly deposited is of one haplotype. Contigs corresponding to the second haplotype have also been deposited. The mitochondrial genome was also assembled and can be found as a contig within the multifasta file of the genome submission.

**Table 1.** Genome data for *Boloria euphrosyne*, ilBolEuph2.2.

Project accession data		
Assembly identifier	ilBolEuph2.2	
Species	*Boloria euphrosyne*	
Specimen	ilBolEuph2	
NCBI taxonomy ID	405023	
BioProject	PRJEB61911	
BioSample ID	SAMEA9700870	
Isolate information	ilBolEuph2, male: whole organism (PacBio DNA sequencing and RNA sequencing) ilBolEuph1, female: whole organism (Hi-C sequencing)	
Assembly metrics *		*Benchmark*
Consensus quality (QV)	63.2	*≥ 50*
*k*-mer completeness	100.0%	*≥ 95%*
BUSCO **	C:99.0%[S:98.7%,D:0.3%], F:0.2%,M:0.8%,n:5,286	*C ≥ 95%*
Percentage of assembly mapped to chromosomes	99.57%	*≥ 95%*
Sex chromosomes	ZZ	*localised homologous pairs*
Organelles	Mitochondrial genome: 15.17 kb	*complete single alleles*
Raw data accessions		
PacificBiosciences Sequel IIe	ERR11435987	
Hi-C Illumina	ERR11439642	
PolyA RNA-Seq Illumina	ERR12321226	
Genome assembly		
Assembly accession	GCA_951802675.2	
*Accession of alternate haplotype*	GCA_951802665.2	
Span (Mb)	400.4	
Number of contigs	81	
Contig N50 length (Mb)	9.2	
Number of scaffolds	55	
Scaffold N50 length (Mb)	13.6	
Longest scaffold (Mb)	19.28	
Genome annotation		
Number of protein-coding genes	19,138	
Number of gene transcripts	19,341	

* Assembly metric benchmarks are adapted from column VGP-2020 of “Table 1: Proposed standards and metrics for defining genome assembly quality” from Rhie *et al.* (2021). ** BUSCO scores based on the lepidoptera_odb10 BUSCO set using version 5.3.2. C = complete [S = single copy, D = duplicated], F = fragmented, M = missing, n = number of orthologues in comparison. A full set of BUSCO scores is available at https://blobtoolkit.genomehubs.org/view/ilBolEuph2_2/dataset/ilBolEuph2_2/busco.

**Table 2.** Chromosomal pseudomolecules in the genome assembly of *Boloria euphrosyne*, ilBolEuph2.

INSDC accession	Chromosome	Length (Mb)	GC%
OX637871.1	1	16.18	33.0
OX637872.1	2	15.95	33.0
OX637873.1	3	15.86	33.0
OX637874.1	4	15.75	33.5
OX637875.1	5	15.44	33.0
OX637876.1	6	14.99	32.5
OX637877.1	7	14.76	32.5
OX637878.1	8	14.73	33.0
OX637879.1	9	14.45	32.5
OX637880.1	10	14.21	33.0
OX637881.1	11	14.08	32.5
OX637882.1	12	13.75	33.0
OX637883.1	13	13.61	33.0
OX637884.1	14	13.37	32.5
OX637885.1	15	13.22	33.0
OX637886.1	16	12.98	33.0
OX637887.1	17	12.94	33.0
OX637888.1	18	12.44	33.0
OX637889.1	19	12.4	33.5
OX637890.1	20	12.05	33.0
OX637891.1	21	11.62	33.0
OX637892.1	22	10.85	33.5
OX637893.1	23	10.76	33.5
OX637894.1	24	10.56	33.0
OX637895.1	25	9.62	35.5
OX637896.1	26	9.41	33.5
OX637897.1	27	9.2	33.0
OX637898.1	28	8.86	34.5
OX637899.1	29	8.28	35.5
OX637900.1	30	7.17	34.5
OX637870.1	Z	19.28	32.5
OY041595.1	MT	0.02	19.5

The estimated Quality Value (QV) of the final assembly is 63.2 with *k*-mer completeness of 100.0%, and the assembly has a BUSCO v5.3.2 completeness of 99.0% (single = 98.7%, duplicated = 0.3%), using the lepidoptera_odb10 reference set ( *n* = 5,286).

Metadata for specimens, barcode results, spectra estimates, sequencing runs, contaminants and pre-curation assembly statistics are given at https://links.tol.sanger.ac.uk/species/405023.

## Genome annotation report

The *Boloria euphrosyne* genome assembly (GCA_951802675.2) was annotated at the European Bioinformatics Institute (EBI) on Ensembl Rapid Release. The resulting annotation includes 19,341 transcribed mRNAs from 19,138 protein-coding genes ( Table 1; https://rapid.ensembl.org/Boloria_euphrosyne_GCA_951802675.2/Info/Index).

## Methods

### Sample acquisition and nucleic acid extraction

*Boloria euphrosyne* specimens were collected from Glasdrum National Nature Reserve, Scotland, UK (latitude 56.57, longitude -–5.23) on 2021-06-01 by hand netting. A male specimen (specimen ID SAN0001687, ToLID ilBolEuph2) was used for DNA sequencing, and a female specimen (specimen ID SAN0001680, ToLID ilBolEuph1) was used for Hi-C sequencing. The specimens were collected by Konrad Lohse, Sam Ebdon, Alex Mackintosh and Simon Martin (University of Edinburgh) and identified by Konrad Lohse, and frozen from live at –80 °C.

The workflow for high molecular weight (HMW) DNA extraction at the Wellcome Sanger Institute (WSI) Tree of Life Core Laboratory includes a sequence of core procedures: sample preparation; sample homogenisation, DNA extraction, fragmentation, and clean-up. In sample preparation, the ilBolEuph2 sample was weighed and dissected on dry ice ( Jay *et al.*, 2023).

Tissue from the whole organism was homogenised using a PowerMasher II tissue disruptor ( Denton *et al.*, 2023a). HMW DNA was extracted using the Automated MagAttract v2 protocol ( Oatley *et al.*, 2023a). DNA was sheared into an average fragment size of 12–20 kb in a Megaruptor 3 system with speed setting 31 ( Bates *et al.*, 2023). Sheared DNA was purified by solid-phase reversible immobilisation ( Oatley *et al.*, 2023b): in brief, the method employs a 1.8X ratio of AMPure PB beads to sample to eliminate shorter fragments and concentrate the DNA. The concentration of the sheared and purified DNA was assessed using a Nanodrop spectrophotometer and Qubit Fluorometer and Qubit dsDNA High Sensitivity Assay kit. Fragment size distribution was evaluated by running the sample on the FemtoPulse system.

RNA was extracted from remaining tissue of ilBolEuph2 in the Tree of Life Laboratory at the WSI using the RNA Extraction: Automated MagMax™ *mir*Vana protocol (do Amaral *et al.*, 2023). The RNA concentration was assessed using a Nanodrop spectrophotometer and a Qubit Fluorometer using the Qubit RNA Broad-Range Assay kit. Analysis of the integrity of the RNA was done using the Agilent RNA 6000 Pico Kit and Eukaryotic Total RNA assay.

Protocols developed by the WSI Tree of Life laboratory are publicly available on protocols.io ( Denton *et al.*, 2023b).

### Sequencing

Pacific Biosciences HiFi circular consensus DNA sequencing libraries were constructed according to the manufacturers’ instructions. Poly(A) RNA-Seq libraries were constructed using the NEB Ultra II RNA Library Prep kit. DNA and RNA sequencing was performed by the Scientific Operations core at the WSI on Pacific Biosciences Sequel IIe (HiFi) and Illumina NovaSeq 6000 (RNA-Seq) instruments. Hi-C data were also generated from whole organism tissue of ilBolEuph1 using the Arima2 kit and sequenced on the Illumina NovaSeq 6000 instrument.

### Genome assembly, curation and evaluation

Assembly was carried out with Hifiasm ( Cheng *et al.*, 2021) and haplotypic duplication was identified and removed with purge_dups ( Guan *et al.*, 2020). The assembly was then scaffolded with Hi-C data ( Rao *et al.*, 2014) using YaHS ( Zhou *et al.*, 2023). The assembly was checked for contamination and corrected using the gEVAL system ( Chow *et al.*, 2016) as described previously ( Howe *et al.*, 2021). Manual curation was performed using gEVAL, HiGlass ( Kerpedjiev *et al.*, 2018) and PretextView ( Harry, 2022). The mitochondrial genome was assembled using MitoHiFi ( Uliano-Silva *et al.*, 2023), which runs MitoFinder ( Allio *et al.*, 2020) or MITOS ( Bernt *et al.*, 2013) and uses these annotations to select the final mitochondrial contig and to ensure the general quality of the sequence.

A Hi-C map for the final assembly was produced using bwa-mem2 ( Vasimuddin *et al.*, 2019) in the Cooler file format ( Abdennur & Mirny, 2020). To assess the assembly metrics, the *k*-mer completeness and QV consensus quality values were calculated in Merqury ( Rhie *et al.*, 2020). This work was done using Nextflow ( Di Tommaso *et al.*, 2017) DSL2 pipelines “sanger-tol/readmapping” ( Surana *et al.*, 2023a) and “sanger-tol/genomenote” ( Surana *et al.*, 2023b). The genome was analysed within the BlobToolKit environment ( Challis *et al.*, 2020) and BUSCO scores ( Manni *et al.*, 2021; Simão *et al.*, 2015) were calculated.

Table 3 contains a list of relevant software tool versions and sources.

**Table 3.** Software tools: versions and sources.

Software tool	Version	Source
BlobToolKit	4.2.1	https://github.com/blobtoolkit/blobtoolkit
BUSCO	5.3.2	https://gitlab.com/ezlab/busco
gEVAL	N/A	https://geval.org.uk/
Hifiasm	0.16.1-r375	https://github.com/chhylp123/hifiasm
HiGlass	1.11.6	https://github.com/higlass/higlass
Merqury	MerquryFK	https://github.com/thegenemyers/MERQURY.FK
MitoHiFi	3	https://github.com/marcelauliano/MitoHiFi
PretextView	0.2	https://github.com/wtsi-hpag/PretextView
purge_dups	1.2.5	https://github.com/dfguan/purge_dups
sanger-tol/genomenote	v1.0	https://github.com/sanger-tol/genomenote
sanger-tol/readmapping	1.1.0	https://github.com/sanger-tol/readmapping/tree/1.1.0
YaHS	1.2a.2	https://github.com/c-zhou/yahs

### Genome annotation

The BRAKER2 pipeline ( Brůna *et al.*, 2021) was used in the default protein mode to generate annotation for the *Boloria euphrosyne* assembly (GCA_951802675.2) in Ensembl Rapid Release at the EBI.

### Wellcome Sanger Institute – Legal and Governance

The materials that have contributed to this genome note have been supplied by a Darwin Tree of Life Partner. The submission of materials by a Darwin Tree of Life Partner is subject to the **‘Darwin Tree of Life Project Sampling Code of Practice’**, which can be found in full on the Darwin Tree of Life website here. By agreeing with and signing up to the Sampling Code of Practice, the Darwin Tree of Life Partner agrees they will meet the legal and ethical requirements and standards set out within this document in respect of all samples acquired for, and supplied to, the Darwin Tree of Life Project.

Further, the Wellcome Sanger Institute employs a process whereby due diligence is carried out proportionate to the nature of the materials themselves, and the circumstances under which they have been/are to be collected and provided for use. The purpose of this is to address and mitigate any potential legal and/or ethical implications of receipt and use of the materials as part of the research project, and to ensure that in doing so we align with best practice wherever possible. The overarching areas of consideration are:

•     Ethical review of provenance and sourcing of the material

•     Legality of collection, transfer and use (national and international)

Each transfer of samples is further undertaken according to a Research Collaboration Agreement or Material Transfer Agreement entered into by the Darwin Tree of Life Partner, Genome Research Limited (operating as the Wellcome Sanger Institute), and in some circumstances other Darwin Tree of Life collaborators.

## Data Availability

European Nucleotide Archive: *Boloria euphrosyne* (pearl-bordered fritillary). Accession number PRJEB61911; https://identifiers.org/ena.embl/PRJEB61911 ( Wellcome Sanger Institute, 2023). The genome sequence is released openly for reuse. The *Boloria euphrosyne* genome sequencing initiative is part of the Darwin Tree of Life (DToL) project. All raw sequence data and the assembly have been deposited in INSDC databases. Raw data and assembly accession identifiers are reported in Table 1.

## Author information

Members of the Wellcome Sanger Institute Tree of Life Management, Samples and Laboratory team are listed here: https://doi.org/10.5281/zenodo.10066175.

Members of Wellcome Sanger Institute Scientific Operations: Sequencing Operations are listed here: https://doi.org/10.5281/zenodo.10043364.

Members of the Wellcome Sanger Institute Tree of Life Core Informatics team are listed here: https://doi.org/10.5281/zenodo.10066637.

Members of the Tree of Life Core Informatics collective are listed here: https://doi.org/10.5281/zenodo.5013541.

Members of the Darwin Tree of Life Consortium are listed here: https://doi.org/10.5281/zenodo.4783558.
