# Supplementary material for: The genome sequence of the Pearl-bordered Fritillary, Boloria euphrosyne (Linnaeus, 1758)
Source: Wellcome Open Res. 2024 May 15;9:267. [Version 1] doi: 10.12688/wellcomeopenres.21586.1 (PMC11462119; doi:10.12688/wellcomeopenres.21586.1)
Supplement: Peer review history

# Reviewer report: Reviewer response for version 1

**DOI:** 10.21956/wellcomeopenres.23858.r100381

**Published:** 2024-10-08

**Authors:** Casper Breuker

**Affiliations:** 1 Oxford Brookes University, Oxford, England, UK

The background is well-written, but only the final sentence provides some form of "justification" as to what the value of genomic resources for this species is. Yes, that single sentence summarizes it (what kind of "diversity of adaptation" did the authors have in mind?), but it would have been nice to have more of a narrative in the background as to how such genomic/genetic resources may be of value. For example, it would be great to see some genetic narrative coupled to a paper like this: Blomfield A *et al*.(2023) ^1^

http://10.1007/s00442-023-05357-2

Are sufficient details of methods and materials provided to allow replication by others?

Yes

Is the rationale for creating the dataset(s) clearly described?

Partly

Are the datasets clearly presented in a useable and accessible format?

Yes

Are the protocols appropriate and is the work technically sound?

Yes

Reviewer Expertise:

Evolutionary developmental biology, environmental genomics

I confirm that I have read this submission and believe that I have an appropriate level of expertise to confirm that it is of an acceptable scientific standard.

## References

[1] : Population synchrony indicates functional connectivity in a threatened sedentary butterfly. *Oecologia* .2023;201(4) : 10.1007/s00442-023-05357-2 979-989 10.1007/s00442-023-05357-2 36976354 PMC10113297

# Reviewer report: Reviewer response for version 1

**DOI:** 10.21956/wellcomeopenres.23858.r98121

**Published:** 2024-09-25

**Authors:** Arun Arumugaperumal

**Affiliations:** 1 Department of Biotechnology, Rajalakshmi Engineering College, Chennai, Tamilnadu, India

The authors have presented the genome sequence of the butterfly Boloria euphrosyne. It is commonly known as Pearl-bordered Fritillary. The assembly reported herewith was of size 400.4 megabases long. The genome was scaffolded into 31 chromosomes. The mitochondrial genome was also sequenced. A total of 19,138 genes were identified in the genome assembly. Annotation was done using Ensembl repository.

 Based on a google scholar article search, this was the first report of the whole genome of this individual. The photograph of the specimen wings is of good quality. The scale provided in the image adds to the size approximation for readers. The genome assembly size of 400 Mbp size goes in hand with other similar species sequenced ( *Boloria selene*). 65X coverage in PacBio sequencing technology was used to get high quality genome sequence.

 BUSCO analysis shows the completeness of a genome sequence. For this species there is a completeness percentage of 99% indicating that the genome assembly is a near complete one. 

 Since a closely related species genome was already published, the authors could have tried to include a comparison plot.

Are sufficient details of methods and materials provided to allow replication by others?

Yes

Is the rationale for creating the dataset(s) clearly described?

Yes

Are the datasets clearly presented in a useable and accessible format?

Yes

Are the protocols appropriate and is the work technically sound?

Yes

Reviewer Expertise:

Bioinformatics, Genomics

I confirm that I have read this submission and believe that I have an appropriate level of expertise to confirm that it is of an acceptable scientific standard.
